# Effect of Nephrology Care on Mortality in Incident Dialysis Patients: A Population-Based Cohort Study

**DOI:** 10.3390/jpm11111071

**Published:** 2021-10-24

**Authors:** Cheng-Yin Chung, Ping-Hsun Wu, Yi-Wen Chiu, Shang-Jyh Hwang, Ming-Yen Lin

**Affiliations:** 1 Division of Nephrology, Department of Internal Medicine, Ministry of Health and Welfare, Pingtung Hospital, Pingtung 90054, Taiwan; 950085@kmuh.org.tw; 2 Division of Nephrology, Department of Internal Medicine, Kaohsiung Medical University Hospital, Kaohsiung Medical University, Kaohsiung 80756, Taiwan; 970392kmuh@gmail.com (P.-H.W.); chiuyiwen@kmu.edu.tw (Y.-W.C.); sjhwang@kmu.edu.tw (S.-J.H.); 3 Graduate Institute of Medicine, College of Medicine, Kaohsiung Medical University, Kaohsiung 80756, Taiwan; 4 Department of Renal Care, College of Medicine, Kaohsiung Medical University, Kaohsiung 80756, Taiwan; 5 Institute of Population Health Sciences, National Health Research Institutes, Miaoli 35053, Taiwan

**Keywords:** dialysis, end-stage kidney disease, mortality, nephrology care, causal inference

## Abstract

Long-term and continuous nephrology care effects on post-dialysis mortality remain unclear. This study aims to systematically explore the causal effect of nephrology care on mortality for patients with dialysis initiation. We conducted a retrospective cohort study to include incident patients with dialysis for ≥ 3 months in Taiwan from 2004 through 2011. The continuous nephrology care of incident patients in the three years before their dialysis was measured every six months. Continuous nephrology care was determined by 0–6, 0–12, …, 0–36 months and their counterparts; and none, intermittent, 0–6 months, …, and 0–36 months. Simple and weighted hazards ratio (HR) and 95% confidence interval (CI) for one-year mortality were estimated after propensity score (PS) matching. We included a total of 44,698 patients (mean age 63.3 ± 14.2, male 51.9%). Receiving ≥ 1 year predialysis nephrology care was associated with a 22% lower post-dialysis mortality hazard. No different effects were found (ranges of PS matching HR: 0.77–0.80) when comparing the defined duration of nephrology care with their counterparts. Stepped survival benefits were newly identified in the intermittent care, which had slightly lower HRs (weighted HR: 0.88, 95% CI: 0.79–0.97), followed by reviving care over six months to two years (ranges of weighted HR: 0.60–0.65), and reviving care over two years (ranges of weighted HR: 0.48–0.52). There was no existing critical period of nephrology care effect on post-dialysis, but there were extra survival benefits when extending nephrology care to >2 years, which suggests that continuous and long-term care during pre-dialysis/chronic kidney disease phase is required.

## 1. Introduction

Globally, over 850 million people with chronic kidney disease (CKD) need appropriate management and care, to improve risks of adverse outcomes and elevate their quality of care [1]. Several chronic conditions usually accompany CKD and may affect consequent patient care and outcomes. For example, hypertension accounts for about 60% to 90% of the global CKD population [2], which is the main comorbidity of CKD. Diabetic nephropathy affects approximately 20%–40% of patients who have diabetes mellitus and also contributes to a large proportion of end-stage kidney disease (ESKD) [3]. Chronic conditions in CKD were also associated with whether patients can receive appropriate care when their disease requires it [4]. Without appropriate management and control, these chronic conditions could incur the rapid progression of renal dysfunction and attribute more risks of developing cardiovascular morbidity and mortality [5]. 

Although results from previous studies mostly support the notion that nephrology care during the pre-ESKD phase may be associated with survival benefits after dialysis is initiated [6,7,8], optimal nephrology care intervals, visits, and contents could be influenced by reimbursement policy and are yet to be determined. In general, timely nephrology referral, regular nephrology care visits, and multidisciplinary care were suggested when patients’ glomerular filtration rate reached 30 mL/min/1.73 m^2^ or below, or for patients facing uncontrollable renal disease deterioration [9,10]. However, arbitrarily categorizing the timing of nephrology referral as early or late, and determining nephrology care in short time windows, may lose accuracy in quantifying the effects of nephrology care on patient benefits after dialysis. It is worthwhile to conduct a study to define nephrology care by longer durations before dialysis in a less restrictive reimbursement system for nephrology care. 

The current study systematically determined exposure to nephrology care by six-monthly intervals until the three years prior to dialysis, and comprehensively explored the average causal effects of nephrology care on post-dialysis mortality using the propensity score (PS) approach. 

## 2. Materials and Methods

### 2.1. Study Design and Population 

We conducted a retrospective population-based cohort study to assess the effect of continuous nephrology care during the pre-dialysis CKD status on one-year mortality after dialysis. All adult patients who initiated long-term (≥3 months) dialysis therapy (hemodialysis or peritoneal dialysis) between 2007 to 2011 were identified from the Taiwan National Health Insurance Research Database, Ministry of Health and Welfare (NHIRD_MOHW). Taiwan NHIRD is a population-based claim database containing 23 million people’s details of clinical information (including the date, expenditures, and diagnosis related to both inpatient and outpatient procedures; prescription details; examinations; and operations) that has been well applied in epidemiological and clinical research [11,12]. These databases are maintained and managed by Health and Welfare Data Science Center (MOHW) and have been opened to the researcher through on-site analysis with a remote connection to Taiwan’s Ministry of Health and Welfare server after formal application. We identified patients who initiated long-term dialysis based on the definitions published in our previous studies^4^. In brief, dialysis patients were identified by several International Classification of Disease, Ninth Revision, Clinical Modification, (ICD-9-CM) codes from the Registry of Catastrophic Illness dataset and ensured those who underwent dialysis ≥3 months by dialysis reimbursed codes (Supplementary Table S1 online).

### 2.2. Nephrology Care

We determined nephrology care by specialty of medical visit which could be obtained from outpatient claim data. The inspection time window of nephrology care was selected for every 6-monthly interval, retrospectively from the maintenance dialysis to three years prior. The continuous nephrology care within each 6-monthly inspection interval was defined by at least two nephrology visits, and the longest nephrology visit interval within each time-windows ≥3 months. Based on the above definition, we classified the patients with or without continuous nephrology care at each setting time six-month interval and their counterparts, to determine the crucial period of nephrology care. Furthermore, we detected cumulative continuous nephrology care retrospectively from dialysis by classifying patients into the exclusive groups: none, intermittent, 0–6 months, 0–12 months (1 year), 0–18 months, 0–24 months, 0–30 months, and 0–36 months. For example, patients in the 0–6 months group were required to have at least two nephrology visits within six months before dialysis, with the longest nephrology visit interval ≥3 months, to be called continuous nephrology care. Patients in the 0–12 months group were required to have both continuous nephrology care in 0–6 and 7–12 months before dialysis, while patients in the 0–18 months group were required to have continuous nephrology care in 0–6, 7–12, and 12–18 months, and so on.

### 2.3. Comorbidity Assessment

Because patients’ comorbidities mainly determine patterns of nephrology care, we defined 29 comorbidities that can be obtained with high positive predicted values (>70%) through ICD-9 CM codes from claim data [13]. The 29 comorbidities contain: atrial fibrillation, chronic heart failure, diabetes, hypertension, peripheral vascular disease, stroke or transient ischemic attack, asthma, lymphoma, metastatic cancer, non-metastatic cancer, chronic pulmonary disease, severe constipation, dementia, inflammatory bowel disease, rheumatoid arthritis, alcohol misuse, chronic pain, depression, schizophrenia, chronic viral hepatitis B, cirrhosis, epilepsy, hypothyroidism, irritable bowel syndrome, multiple sclerosis, myocardial infarction, Parkinson’s disease, peptic ulcer disease, and psoriasis (Supplementary Table S2 online). To further evaluate the associations of continuous nephrology care with mortality in various types of comorbidities, we grouped these comorbidities into four main categories based on their pathophysiological similarity and potential for co-management [14,15,16]. 

### 2.4. Death and Other Covariates 

Death after dialysis was determined by the discharge status of last admission hospitalization (death, against advising discharge and discharge under critical conditions), and there were no medical records one year after the last admission date. We also collected information on patient characteristics, namely: age, sex (male or female), insurance amount [fixed premium or dependent, <20,000, 20,000–39,999, and ≥39,999 New Taiwan dollars (~1333 US dollars)], region (north, center, south, and east), urbanization of residence (urban or rural), Charlson comorbidity index score, and potential confounding drug prescriptions (anticoagulation, antiplatelet, or antidiabetic agents, insulin, steroid, and non-steroidal anti-inflammatory drugs). All these data were derived from outpatient claims by ATC codes (Supplementary Table S3 online). Charlson comorbidity index scores were calculated based on diseases and the formula reported in a previous study [17], while prescribing listing confounding medicines over 90 days within 3 years before dialysis was considered per user. 

### 2.5. Statistical Analysis

We mainly displayed the group of ≥1 year continuous nephrology care and its counterparts to reflect the associations of covariates with receiving continuous nephrology care. Continuous and categorical data were described by mean ± standard deviation or median (interquartile range) and percentage, respectively. The differences of patient characteristics between groups were evaluated by independent t-tests for continuous variables and χ^2^ tests for categorical variables. PS approaches were adopted to estimate the average causal effect of nephrology care on mortality. The estimated PSs of different definitions of nephrology care were generated by multiple logistic and nominal regression models that were developed through forced entering selected covariates. One-to-one greedy, with exact sex and without replacement, a 0.2 caliper distance, the PS matching approach was applied for groups of receiving or not receiving nephrology care, and the balance of each covariate after matching by the standardized difference was assessed. The average cumulative nephrology effect was classified by the eight different groups in the population using different weights to balance all covariate distributions. We further inspected the relationships between the main factors, age and number of comorbidities, and the cumulative nephrology care by heat map. The one-year mortality proportions after dialysis were described by the Kaplan Meier approach, and the differences between groups assessed by the Gehan–Breslow–Wilcoxon test. Due to violating proportional hazard assumption, the interaction term of time and nephrology care was applied in the Cox proportional hazards regression model. The effect of nephrology care on mortality at one year after dialysis started was displayed by hazard ratio (HR) and 95% confidence interval (CI). All statistical operations were performed using SAS (version 9.4, SAS Institute, Cary, NC, USA) and the mnps function in the twang package of R software (version 4.0, Taipei, Taiwan) [18]. 

## 3. Results

### 3.1. Patient Characteristics 

**Figure 1:**
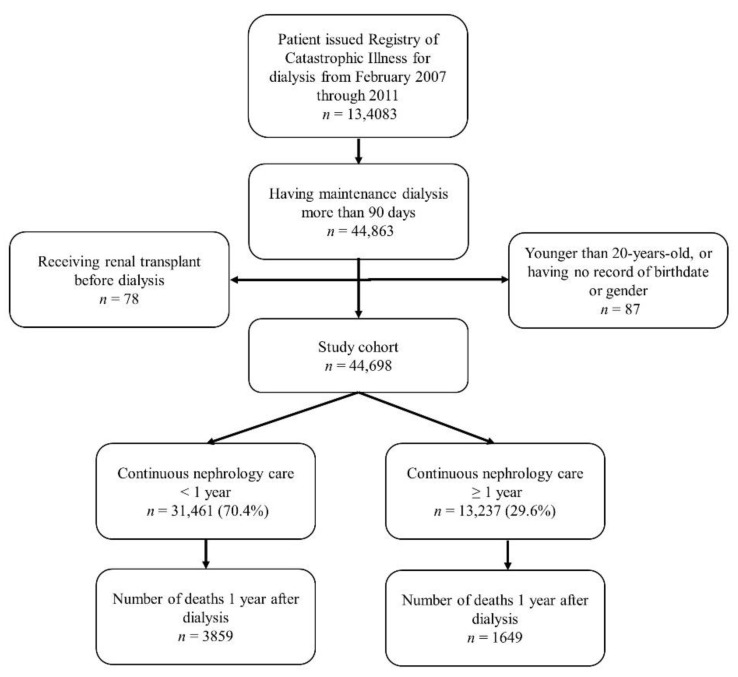
Study design flow chart.

To maintain study validity, we adopted the strict criteria to include our study subjects from databases and excluded all those who had ever received renal transplantation, were not adults, or lacked relevant information (Figure 1). 

Among the study patients (*n* = 44,698), only nearly 30% of patients with CKD received nephrology care for more than one year. Compared to patients without regular nephrology care, patients with nephrology care time ≥1 year were significantly more likely to be of old age (64.8 ± 13.3 vs. 62.7 ± 14.5 years, *p* < 0.001), females (53.2 vs. 42.0%, *p* < 0.001), living north, in an urban region, and with higher income, greater severity of diseases, more steroid prescriptions, but with fewer antidiabetic agents and NSAIDs prescriptions (Table 1). Compatible baseline characteristics between the groups with a range of standardized differences (−0.05, 0.01) were achieved after carrying out PS matching. 

### 3.2. Comorbidity and Nephrology Care 

CKD in patients is mainly accompanied by hypertension (52.9%), diabetes (48.5%), and chronic pain (26.2%) (Table 2). A significantly higher prevalence of chronic heart failure (16.5% vs. 9.6%, *p* < 0.001), diabetes (51.4% vs. 41.6%, *p* < 0.001), hypertension (53.9% vs. 50.4%, *p* < 0.001), stroke (12.0% vs. 8.1%, *p* < 0.001), asthma (1.9% vs. 1.5%, *p* < 0.001), alcohol misuse (0.5% vs. 0.1%, *p* < 0.001), cirrhosis (2.2% vs. 1.7%, *p* < 0.001), and myocardial infarction (2.7% vs. 1.4%, *p* < 0.001) were observed in the group of non-continuous nephrology care. However, patients who received nephrology care for more than one year present a higher prevalence of non-metastatic cancer (3.0% vs. 2.3%, *p* < 0.001), depression (2.5% vs. 1.9%, *p* < 0.001), chronic hepatitis B (1.3% vs. 1.1%, *p* < 0.05), irritable bowel syndrome (1.3% vs. 1.1%, *p* < 0.05) and peptic ulcer disease (7.8% vs. 6.6%, *p* < 0.001). After PS matching, similar distributions in listed comorbidities between the groups were obtained.

### 3.3. Distribution and Nephrology Care 

**Figure 2:**
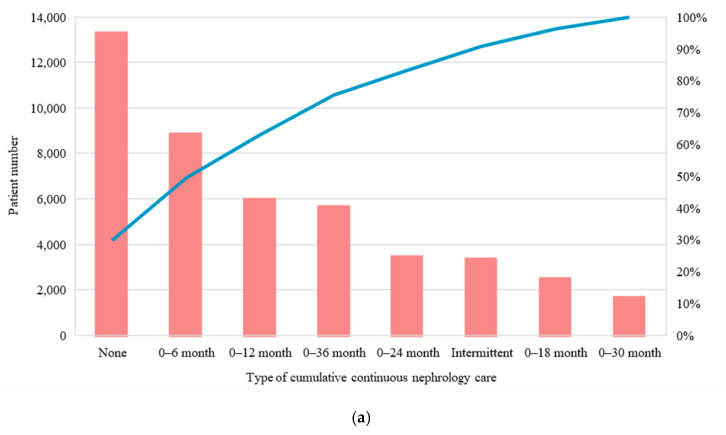

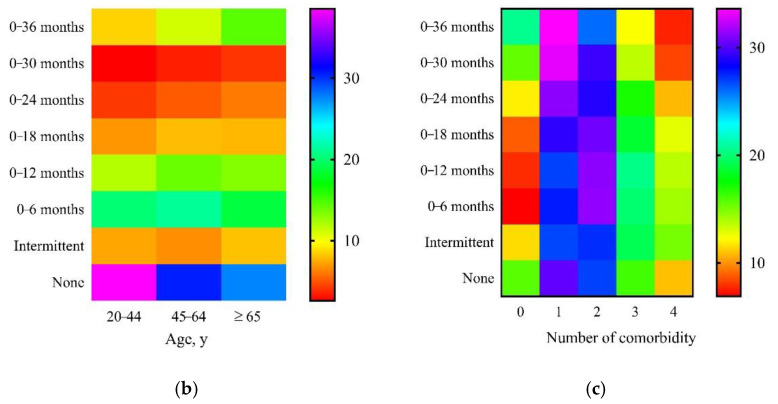
Distribution in nephrology care. (**a**) Patient number in different types of nephrology care and their cumulative percentage; (**b**) Heat map comparing proportions of types of cumulative nephrology care between age groups (20–44, 45–64, and ≥ 65 years); (**c**) Heat map comparing proportions of types of cumulative nephrology care in patients with different comorbidity numbers (0, 1, 2, 3, and ≥4).

Nearly half of the study participants did not receive regular nephrology care in the 3-year observational period before dialysis, or only had regular nephrology care until 6 months before dialysis. It is worth noting that a few patients (7.5%) intermittently received nephrology care (Figure 2a). The relationships between age groups, the number of comorbidities, and cumulative nephrology care are presented in the heat map. Young patients (age 20–44 years) showed a greater probability of being in the group of limited nephrology care in the 3-year observational period before dialysis (Figure 2b). Patients who never received or received regular nephrology care ≥30 months have the highest proportion of one comorbidity; whereas those who intermittently received or received regular nephrology care 0–6, 0–12, or 0–18 months have the highest proportion of two comorbidities (Figure 2c).

### 3.4. Nephrology Care and Mortality 

**Figure 3:**
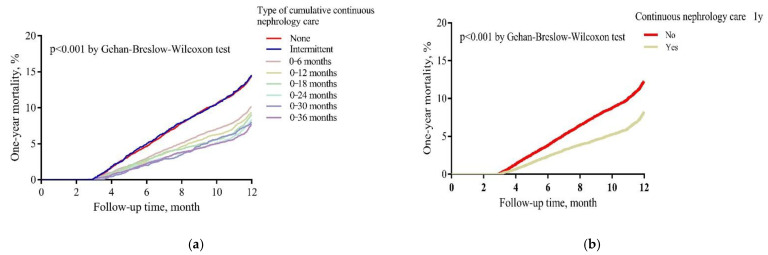
Cumulative mortality proportion between (**a**) ≥1 year vs. <1 year nephrology care; (**b**) types of cumulative nephrology care. The time was calculated from the date of dialysis initiation to the date of death, or the end date of the first year, whichever came first. Cumulative one-year mortality proportions were estimated by the Kaplan Meier approach and assessed the differences between groups by Gehan–Breslow–Wilcoxon test.p<0.001.

The cumulative mortality proportion in the group of continuous nephrology care ≥ 1 year was lower than those in the counter group (8.2% vs. 12.3%; *p* < 0.001 by Gehan–Breslow–Wilcoxon test) (Figure 3a). After dividing by eight exclusive groups, the cumulative mortality proportion was highest in the groups of no care and intermittently receiving nephrology care (14.4 and 14.5%), compared to those in the other groups (Figure 3b). 

**Figure 4:**
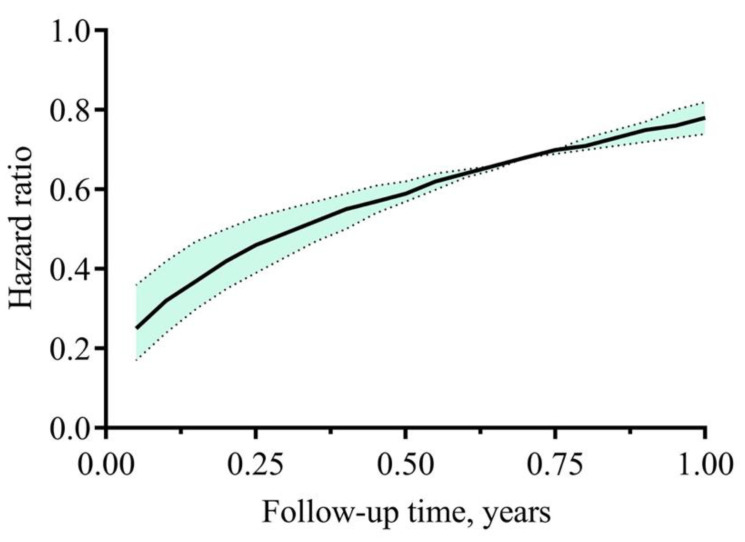
The time-dependent hazard ratio of one-year mortality comparing patients receiving nephrology care ≥1 year with those <1 year. The estimated PSs of receiving nephrology care were generated by multiple logistic regression models that were developed through forced entering selected covariates. One-to-one Greedy, with exact sex and without replacement, a 0.2 caliper distance, the PS matching approach was applied for groups of receiving, or not receiving nephrology care. Cox proportional hazards regression model with added interaction term of time and nephrology care was applied in the matched cohort.

The time-dependent HRs are in Figure 4. The survival benefit for the group of continuous nephrology care ≥1 year before dialysis initiation gradually decreased over time, and an average lower 22% mortality hazard (PS-matching HR: 0.78, 95% CI: 0.74–0.82) compared with those in the group of continuous nephrology care <1 year was demonstrated at the end of one year after the start of dialysis (Figure 4). 

In addition, we observed that the continuous average nephrology care effect on mortality (ranges of PS-matching HR: 0.77–0.80) when we applied different periods (0–6, 0–12, …, 0–36) to determine continuous nephrology care and made matching approaches by PS scores (Table 3).

Compared to patients without nephrology care, a stepped reduction in HR was identified when patients received more nephrology care. Patients who received continuous nephrology care over two years presented the lowest HRs (ranges of weighted HR: 0.48–0.52), followed by patients who received continuous nephrology care from six months to two years (ranges of weighted HR: 0.60–0.65), and patients with intermittent nephrology care (weighted HR: 0.88, 95% CI: 0.79–0.97) (Table 4).

## 4. Discussion

The study systematically identified that patients younger than 45 years or having two comorbidities were associated with limited nephrology care in a 3-year observational period before dialysis. The benefit of nephrology care for mortality was large during dialysis initiation and was followed by a gradual shrinkage trend to an average 22% lower at one year after dialysis. There were no significant differences in the benefit of nephrology care for mortality when we defined continuous nephrology care by different periods. A stepped dose–response effect of nephrology care on mortality was observed, with patients receiving continuous nephrology care for over two years, care for six months to two years, intermittent nephrology care, and no nephrology care, responding progressively worse.

Although timely receipt of nephrology care in CKD was generally suggested by recent nephrology guidelines [10], there is still room to improve the content of nephrology care that influences the mortality in the dialysis population. Liu et al. observed a large patient cohort with CKD stage 4 and found that, once patients had outpatient nephrology visits, this was associated with a 12% lower mortality risk [19], but a slight increase in dialysis probability [20]. Another earlier study finds that late care (<6 months before dialysis), lower cumulative care (≤5 nephrology visits within a 3-year period), and inconsistent critical period care (≥3 of the 6 months before dialysis) are independent factors associated with mortality within one-year after dialysis begins [20]. Yang et al. further combined the concept of early nephrology referral determined by ≥6 months before dialysis and frequency of nephrology visit to demonstrate that a ~10% lower 1-year post-dialysis major adverse cardiovascular events could be observed in patients with early-frequent nephrology care, compared to those with late nephrology-care [6]. Unlike the definitions used in these studies, our approach of determining nephrology care every 6 months until 3 years before dialysis is more objective than others. Furthermore, estimating the average care effects using causal inference methods could also provide new insights in encouraging nephrology care to be implemented as early as 2 years before dialysis, not just at any arbitrary time period.

The optimal frequency of nephrology care has not been suggested previously. We identified that, once patients received intermittent nephrology care, this could bring some benefits upon mortality risk. However, extra survival benefits could occur if they extended nephrology care further. A recent study also supported these findings, emphasizing the need for nephrology care length and consistency for improving major adverse events in post-dialysis periods [6]. The mechanisms under these findings are unknown and should involve characteristics of the patient, disease, care, and their interactions. It is reasonable to speculate that patients with more cumulative nephrology care should get extra benefits from nephrology care through the early detection and treatment of complications, as well as the strict control of ill conditions. In addition, patients may modify their behavior to reflect disease requirement suggestions by a nephrologist. More research on exploring the difference in patient–physician behaviors during pre-ESKD/CKD phases is warranted.

PS-based methods have been widely applied in observational studies to determine the effect size of intervention on outcomes conditions on the similar distributions of covariates between intervention groups. Both PS matching and weighted methods are popularly used to estimate average intervention effects. In our study, we used the PS matching method for two care group comparisons and the weighted approach for unbalanced and multiple care group comparisons. It is worth noting the main conceptual difference between these two methods. The matching approach reflects the average intervention effect only among those who ultimately received intervention, while the weighted one estimates the average intervention effect in the scenario in which every patient within the population was offered intervention [21]. Combined with suggestions from the Kidney Disease: Improving Global Outcomes guidelines [22] and recent findings [23], our findings advocate that patients with CKD should continuously receive nephrology care for at least two years.

Age and comorbidity in CKD were the main factors associated with continuous receipt of nephrology care [24]. Younger CKD patients may be preoccupied with their jobs, causing them to seek nephrology care less frequently. On the other hand, patients who may have more comorbidities may face similar situations, since a physician caring for their primary conditions, (such as hypertension or diabetes mellitus), may be too late to detect CKD until it has progressed to end-stage [4]. The above findings suggest that different care models for these patients should be developed to promote prompt nephrology referral and adherence to nephrology care.

Several advantages of our study are worth pointing out. The single and less restrictive healthcare reimbursement system ensures that most decisions for receiving nephrology care were determined by a physician’s judgment rather than reimbursement policy. Thus, nephrology care in this study was defined by a more practical approach, which enables easier translation of the results into clinical practice. Finally, we adopted more complete methods for defining comorbidity and making a causal inference, validating our findings in a manner similar to results from randomized clinical trials.

Some limitations need to be declared. First, we could not rule out the influences of patient compliance on our inference. It is undeniable that compliant patients may manage their chronic diseases well and lead to better survival. Therefore, interpreting our results should be cautious. Second, the lack of patient behavior and laboratory information prevents us from exploring the mechanisms of nephrology care on mortality. In addition, we could not understand the impacts of medication compliance on our estimation from claim data. However, we believe the influences should be small because physicians had the ability to modify prescriptions at each medical visit when required. Fourth, the study was unable to determine the cause of renal disease. Although they only account for a small proportion of our study subjects, some etiologies can cause rapid renal function progression, resulting in the patients not having the same chance of having nephrology visits as those with slow renal progression. Finally, our patients were from the Taiwanese population covered by the NHI program and derived from older sample data, limiting their generalizability.

## 5. Conclusions

In conclusion, this study demonstrates that there was no critical period whereupon nephrology care produced a bigger effect on post-dialysis mortality, but stepped extra survival benefits were observed when extending nephrology care, which suggests that continuous and long-term nephrology care during the pre-ESKD/CKD phase is required.

## Figures and Tables

**Table 1:** Patient characteristics.

		Before PS Matched		*p*-Value	After PS Matched		Standardized Difference
	Overall	Continuous Nephrology Care ≥ 1 Year			Continuous Nephrology Care ≥ 1 Year		
		No	Yes		No	Yes	
N	44,698	31,461 (70.4)	13,237 (29.6)		13,193	13,193	
Age, year	63.3 ± 14.2	62.7 ± 14.5	64.8 ± 13.3	<0.001	65.0 ± 14.2	64.9 ± 13.4	−0.01
Female sex	21,509 (48.1)	14,467 (46.0)	7042 (53.2)	<0.001	7017 (53.2)	7017 (53.2)	0.0
Geographical region				<0.001			-
North	19,464 (43.6)	13,586 (43.2)	5878 (44.4)		5927 (44.9)	5860 (44.4)	
Central	9850 (22.0)	6978 (22.2)	2872 (21.7)		2905 (22.0)	2862 (21.7)	
South	13,943 (31.2)	9818 (31.2)	4125 (31.2)		4003 (30.3)	4109 (30.2)	
East	1441 (3.2)	1079 (3.4)	362 (2.7)		358 (2.7)	362 (2.7)	
Urbanization level				<0.001			<0.001
Rural	12,642 (28.3)	9214 (29.3)	3428 (25.9)		3394 (25.7)	3423 (26.0)	
Urban	32,056 (71.7)	22,247 (70.7)	9809 (74.1)		9799 (74.3)	9770 (74.1)	
Premium income, NTD				<0.001			-
Dependent	15,820 (35.4)	10,956 (34.8)	4864 (36.7)		4832 (36.6)	4849 (36.8)	
<20,000	9837 (22.0)	7338 (23.3)	2499 (18.9)		2434 (18.5)	2497 (18.9)	
20,000–39,999	16,556 (37.0)	11,607 (36.9)	4949 (37.4)		5049 (38.3)	4,937 (37.4)	
≥40,000	2485 (5.6)	1560 (5.0)	925 (7.0)		878 (6.7)	910 (6.9)	
Charlson index				<0.001			-
0	3168 (7.1)	2852 (9.1)	316 (2.4)		320 (2.4)	316 (2.4)	
1–2	15,061 (33.7)	9995 (31.8)	5066 (38.3)		5098 (38.6)	5051 (38.3)	
3–4	16,149 (36.1)	11,209 (35.6)	4940 (37.3)		4824 (36.6)	4921 (37.3)	
5–6	8737 (19.6)	6250 (19.9)	2487 (18.8)		2510 (19.0)	2478 (18.8)	
≥7	1583 (3.5)	1155 (3.7)	428 (3.2)		441 (3.3)	427 (3.2)	
Confounding drugs							
Anticoagulation agents	626 (1.4)	450 (1.4)	176 (1.3)	0.41	165 (1.25)	175 (1.33)	−0.01
Antiplatelet agents	3893 (8.7)	2767 (8.8)	1126 (8.5)	0.32	1055 (8.0)	1117 (8.5)	−0.02
Antidiabetic agents	16,770 (37.5)	12,721 (40.4)	4049 (30.6)	<0.001	4102 (31.1)	4048 (30.7)	0.01
Insulin	7028 (15.7)	5015 (15.9)	2013 (15.2)	0.05	1985 (15.1)	2010 (15.2)	−0.01
Steroid	2730 (6.1)	1531 (4.9)	1199 (9.1)	<0.001	995 (7.5)	1161 (8.8)	−0.05
NSAIDs	3846 (8.6)	2915 (9.3)	931 (7.0)	<0.001	930 (7.1)	931 (7.1)	<0.001

Abbreviation: NTD, New Taiwan dollar; NSAIDs, non-steroidal anti-inflammatory drugs; PS, propensity score. Data represented as mean ± s.d. for continuous variables and count (percentage) for categorical variables. Different between-group distributions were analyzed using the independent *t* test, and χ^2^ test. *p* value less than 0.05 was considered significant. The propensity score was estimated by a multiple logistic regression model by including all covariates listed in Table 1 and Table 2 as predictors. We performed propensity score one-to-one matching through the Greedy approach and evaluated the balance of covariates between the two nephrology care groups.

**Table 2:** Prevalence of comorbidity by the duration of nephrology care.

		Before PS Matched			After PS Matched		Standardized Difference
	Overall	Continuous Nephrology Care ≥ 1 Year		*p*-Value	Continuous Nephrology Care ≥ 1 Year		
		No	Yes		No	Yes	
N	44,698	31,461 (70.4)	13,237 (29.6)		13,193	13,193	
Concordant comorbidities ^a^							
Atrial fibrillation	238 (0.5)	159 (0.5)	79 (0.6)	0.23	72 (0.5)	79 (0.6)	−0.01
Chronic heart failure	6444 (14.4)	5177 (16.5)	1267 (9.6)	<0.001	1283 (9.7)	1267 (9.6)	<0.001
Diabetes	21,668 (48.5)	16,166 (51.4)	5502 (41.6)	<0.001	5546 (42.0)	5498 (41.7)	0.01
Hypertension	23,643 (52.9)	16,971 (53.9)	6672 (50.4)	<0.001	6778 (51.4)	6655 (50.4)	0.02
Peripheral vascular disease	100 (0.2)	75 (0.2)	25 (0.2)	0.31	24 (0.2)	25 (0.2)	<0.001
Stroke or TIA	4857 (10.9)	3780 (12.0)	1,077 (8.1)	<0.001	1102 (8.4)	1077 (8.2)	0.01
Discordant comorbidities ^a^							
Asthma	779 (1.7)	582 (1.9)	197 (1.5)	0.007	184 (1.4)	197 (1.5)	−0.01
Cancer, lymphoma	97 (0.2)	70 (0.2)	27 (0.2)	0.7	22 (0.2)	27 (0.2)	−0.01
Cancer, metastatic	79 (0.2)	57 (0.2)	22 (0.2)	0.73	19 (0.1)	22 (0.2)	−0.01
Cancer, non-metastatic	1108 (2.5)	713 (2.3)	395 (3.0)	<0.001	376 (2.9)	392 (3.0)	−0.01
Chronic pulmonary disease	2401 (5.4)	1727 (5.5)	674 (5.1)	0.09	611 (4.6)	670 (5.1)	−0.02
Constipation, severe	2062 (4.6)	1441 (4.6)	621 (4.7)	0.6	604 (4.6)	620 (4.7)	−0.01
Dementia	890 (2.0)	624 (2.0)	266 (2.0)	0.86	267 (2.0)	266 (2.0)	<0.001
Inflammatory bowel disease	245 (0.6)	177 (0.6)	68 (0.5)	0.52	60 (0.5)	68 (0.5)	−0.01
Rheumatoid arthritis	828 (1.9)	582 (1.9)	246 (1.9)	0.95	233 (1.8)	246 (1.9)	−0.01
Mental disease and chronic pain ^a^							
Alcohol misuse	169 (0.4)	155 (0.5)	14(0.1)	<0.001	14 (0.1)	14 (0.1)	<0.001
Chronic pain	11,711 (26.2)	8161 (25.9)	3550 (26.8)	0.05	3487 (26.4)	3536 (26.8)	−0.01
Depression	923 (2.1)	587 (1.9)	336 (2.5)	<0.001	305 (2.3)	329 (2.5)	−0.01
Schizophrenia	175 (0.4)	137 (0.4)	38 (0.3)	0.02	32 (0.2)	38 (0.3)	−0.01
Other comorbidities ^a^							
Chronic hepatitis B	513 (1.2)	339 (1.1)	174 (1.3)	0.03	162(1.2)	170 (1.3)	−0.01
Cirrhosis	924 (2.1)	698 (2.2)	226 (1.7)	<0.001	211 (1.6)	225 (1.7)	−0.01
Epilepsy	187 (0.4)	130 (0.4)	57 (0.4)	0.8	56 (0.4)	57 (0.4)	<0.001
Hypothyroidism	210 (0.5)	130 (0.4)	80 (0.6)	0.007	67 (0.5)	78 (0.6)	−0.01
Irritable bowel syndrome	510 (1.1)	332 (1.1)	178 (1.3)	0.009	173 (1.3)	176 (1.3)	<0.001
Multiple sclerosis	62 (0.1)	45 (0.1)	17 (0.1)	0.70	18 (0.1)	17 (0.1)	<0.001
Myocardial infarction	1045 (2.3)	861 (2.7)	184 (1.4)	<0.001	186 (1.4)	184 (1.4)	<0.001
Parkinson’s disease	531 (1.2)	357 (1.13)	174 (1.3)	0.11	172 (1.3)	174 (1.3)	<0.001
Peptic ulcer disease	3103 (6.9)	2072 (6.6)	1031 (7.8)	<0.001	967 (7.3)	1023 (7.8)	−0.02
Psoriasis	258 (0.6)	168 (0.5)	90 (0.7)	0.06	79 (0.6)	87 (0.7)	−0.01

Abbreviations: TIA, transient ischemic attack. ^a^ Comorbidities were classified as concordant, discordant, mental disease and chronic pain, and others based on their pathophysiological similarity and potential for co-management. Data represented as mean ± s.d. for continuous variables and count (percentage) for categorical variables. Different between-group distributions were analyzed using the independent *t* test, and χ^2^ test. *p* value lower than 0.05 was considered significant. Propensity score was estimated by multiple logistic regression model by including all covariates listed in Table 1 and Table 2 as predictors. We performed propensity score one-to-one matching through the Greedy approach and evaluated the balance of covariates between the two nephrology care groups.

**Table 3:** Effects of consistent nephrology care at each time interval on one-year mortality after dialysis.

Time Window before Dialysis, Month	Consistent Nephrology Care	Case, *n*	Number of Death, *n*	Follow-Up Time, Person-Years	Mortality Rate (95% CI), per 1000 Patient-Years	PS-Matching HR (95% CI)
0–6	No	16,636	2401	15,765	152.3 (146.3–158.5)	1.00 [reference]
	Yes	28,062	2546	27,228	93.5 (89.9–97.2)	0.79 (0.75–0.83)
0–12	No	25,478	3298	24,303	135.7 (131.2–140.4)	1.00 [reference]
	Yes	19,220	1649	18,691	88.2 (84.1–92.6)	0.78 (0.74–0.82)
0–18	No	31,461	3859	30,102	128.2 (124.2–132.3)	1.00 [reference]
	Yes	13,237	1088	12,892	84.4 (79.5–89.6)	0.78 (0.74–0.82)
0–24	No	34,918	4172	33,458	124.7 (121.0–128.5)	1.00 [reference]
	Yes	9780	775	9535	81.3 (75.8–87.2)	0.78 (0.73–0.83)
0–30	No	37,390	4379	35,867	122.1 (118.5–125.8)	1.00 [reference]
	Yes	7308	568	7126	79.7 (73.4–86.5)	0.80 (0.74–0.86)
0–36	No	39,050	4512	37,485	120.4 (116.9–123.9)	1.00 [reference]
	Yes	5648	435	5508	79.0 (71.9–86.8)	0.77 (071–0.84)

**Table 4:** Effects of cumulative continuous nephrology care on one-year mortality after dialysis.

Type of Continuous Nephrology Care	Case, *n*	Number of Deaths, *n*	Follow-Up Time, Person-Year	Mortality Rate (95% CI), per 1000 Patient-Years	Weighted HR (95% CI)
None	13,297	1916	12,595	152.1 (145.5–159.1)	1.00 [reference]
Intermittent	3339	485	3160	153.5 (140.4–167.8)	0.88 (0.79–0.97)
0–6 month	8842	897	8531	105.1 (98.5–112.3)	0.65 (0.60–0.71)
0–12 month	5983	561	5795	96.8 (89.1–105.2)	0.60 (0.54–0.66)
0–18 month	3457	313	3354	93.3 (83.5–104.3)	0.60 (0.52–0.69)
0–24 month	2472	207	2407	86.0 (75.1–98.6)	0.48 (0.41–0.57)
0–30 month	1660	133	1617	82.3 (69.4–97.5)	0.50 (0.41–0.60)
0–36 month	5648	435	5504	79.0 (71.9–86.8)	0.52 (0.46–0.59)

Abbreviation: CI, confidence interval; HR, hazard ratio. The mortality rate was calculated by the number of deaths divided by the follow-up time and its 95% CI was estimated by Poisson regression model. The propensity score for continuous nephrology care within 0–36 month time-window was estimated by a multiple nominal regression model that was developed through forced entering variables including age, sex, urbanization level, premium income, Charlson index, type of presenting selected comorbidity (concordant, disconcordant, mental, and others), and confounding drugs (anticoagulation agents, antiplatelet agents, antidiabetic agents, insulin, steroid, and non-steroidal anti-inflammatory drugs). Inverse probability weighted for each patient was estimated by the twang package of R software [18].

## Data Availability

The datasets presented in this article are not readily available because these data used in our study should be acquired through formal application to the Health and Welfare Data Science Center, Department of Statistics, Ministry of Health and Welfare, Taiwan. Requests to access the datasets should be directed to https://dep.mohw.gov.tw/DOS/np-2497-113.html. Accessed on 21 September 2021.

## Supplementary Materials

The following are available online at https://www.mdpi.com/article/10.3390/jpm11111071/s1, Table S1: The reimbursement codes for clinical treatment provided by the Taiwan National Health Insurance, Table S2: Comorbidity and corresponding ICD-9-CM codes, Table S3: Anatomical Therapeutic Chemical codes of drugs used concomitantly by patients during the study period.
