# Supplementary material for: Effect of Nephrology Care on Mortality in Incident Dialysis Patients: A Population-Based Cohort Study
Source: J Pers Med. 2021 Oct 24;11(11):1071. doi: 10.3390/jpm11111071 (PMC8622450; doi:10.3390/jpm11111071)
Supplement: Supplementary file 1 [file jpm-11-01071-s001.zip › jpm-1412918-supplementary.pdf]

**Table S1**. The reimbursement codes for clinical treatment provided by the Taiwan National Health Insurance

| Registry or clinical service | Database # | Corresponding reimbursement codes$ |
|---|---|---|
| Catastrophic Illness dataset | Health08 | 585, 403.01, 403.11, 403.91, 404.02, 404.03, 404.12, 404.13, 404.92, 404.93 |
| Hemodialysis treatment per patient visit | Heath04 | 58001C, 58019C, 58020C, 58021C, 58022C, 58023C, 58024C, 58025C, 58027C, 58029C |
| Peritoneal dialysis care | Health04 | 58002C, 58009A, 58009B, 58010A, 58010B, 58011A, 58011AB, 58011B, 58011C, 58012A, 58012B, 58017B, 58017C, 58026C, 58028C |

#Data sources were from and Health04 (Details of ambulatory care orders),. Detailed information can be accessed at https://dep.mohw.gov.tw/dos/lp-3147-113.html

$Information can be accessed at http://www.nhi.gov.tw/query/query2.aspx?menu=20&menu_id=712&WD_ID=830

**Table S2**. Comorbidity and corresponding ICD-9-CM codes

| Type of comorbidity | ICD-9-CM diagnostic code |
|---|---|
| **Concordant comorbidities** | |
| Atrial fibrillation | 427.3 |
| Chronic heart failure | 398.91, 402.01, 402.11, 402.91, 404.01, 404.03, 404.11, 404.13, 404.91, 404.93, 425.4-425.9, 428 |
| Diabetes | 250 |
| Hypertension | 401-405 |
| Peripheral vascular disease | 440.2 |
| Stroke or transient ischemic attack | 362.3, 430, 431, 433.x1, 434.x1, 435, 436 |
| **Discordant comorbidities** | |
| Asthma | 493 |
| Cancer, lymphoma | 200-202, 203.0, 238.6 |
| Cancer, metastatic | 196-199 |
| Cancer, non-metastatic | 153-154, 162-163, 174, 180, 185, 230.3-230.6, 231.2, 233.0-233.1, 233.4 |
| Chronic pulmonary disease | 416.8, 416.9, 490-492, 494-505, 506.4, 508.1, 508.8 |
| Severe constipation, | 560.1, 560.30, 560.39, 560.9, 564.0, 569.83, 569.89 |
| Dementia | 290, 294.1, 331.2 |
| Inflammatory bowel disease | 555, 556 |
| Rheumatoid arthritis | 446.5, 710.0-710.4, 714.0-714.2, 714.8, 725 |
| **Mental disease/Chronic pain** | |
| Alcohol misuse | 265.2, 291.1- 291.3, 291.5-291.9, 303.0, 303.9, 305.0, 357.5, 425.5, 535.3, 571.0-571.3, 980, V11.3 |
| Chronic pain | 307.80, 307.89, 338.0, 338.2, 338.4, 719.41, 719.45-719.47, 719.49, 720.0, 720.2, 720.9, 721.0-721.4, 721.6, 721.8, 721.9, 722, 723.0, 723.1, 723.3-723.9, 724.0-724.6, 724.70, 724.79, 724.8, 724.9, 729.0-729.2, 729.4, 729.5 |
| Depression | 296.2, 296.3, 296.5, 300.4, 309, 311 |
| Schizophrenia | 295 |
| **Other comorbidities** | |
| Chronic viral hepatitis B | 70.2-70.3 |
| Cirrhosis | 571.2, 571.5, 571.6 |
| Epilepsy | 345 |

| | |
|---|---|
| Hypothyroidism | 240.9, 243, 244, 246.1, 246.8 |
| Irritable bowel syndrome | 564.1 |
| Multiple sclerosis | 323, 340, 341.0, 341.9, 377.3 |
| Myocardial infarction | 410 |
| Parkinson's disease | 332 |
| Peptic ulcer disease | 531.7, 531.9, 532.7, 532.9, 533.7, 533.9, 534.7, 534.9 |
| Psoiasis | 696.1 |

Abbreviation: ICD-9-CM, International Classification of Disease, 9th Revision, Clinical Modification. The approaches defining listed comorbidities from claim data were based on previous study that have been proved them with good positive predictive value [1].

**Table S3**. Anatomical Therapeutic Chemical codes of drugs used concomitantly by patients during the study period

| Drug type | ATC classification system codes | Drug name |
|---|---|---|
| Anticoagulation agents | B01AF02, B01AE07, B01AF01, B01AF03, B01AA03 | Apixaban, Dabigatran, Rivaroxaban, Edoxaban, Warfarin |
| Antiplatelet agents | N02BA01, B01AC24, B01AC04 B01AC22 | Aspirin, Ticagrelor, Clopidogrel, Prasugrel |
| Antidiabetic agents | | |
| Oral agents | A10BB09,A10BB12, A10BB07, A10BB01, A10BG03 | Gliclazide, Glimepiride, Glipizide, Glibenclamide, Pioglitazone |
| Insulin | A10AB05, A10AD05, A10AE05, A10AE04 | Insulin Aspart, Insulin Aspart Protamine, Insulin detemir, Insulin Glargine |
| Steroid | H02AB06, H02AB04, H02AB02, H02AB09 | Prednisolone, Methylprednisolone, Dexamethasone, Hydrocortisone |
| Non-steroidal anti-inflammatory drugs | M01AB08, M01AB05, M01AG01, M01AH05, M01AH01 | Etodolac, Diclofenac, Mefenamic acid, Etoricoxib, Celecoxib |

**Table S4**. Checklist of STROBE items of the cohort study

| | Item No | Recommendation | Page No |
|---|---|---|---|
| **Title and abstract** | 1 | (*a*) Indicate the study's design with a commonly used term in the title or the abstract<br>(*b*) Provide in the abstract an informative and balanced summary of what was done and what was found | 1 |
| **Introduction** | | | |
| Background/rationale | 2 | Explain the scientific background and rationale for the investigation being reported | 1&2 |
| Objectives | 3 | State specific objectives, including any prespecified hypotheses | 2 |
| **Methods** | | | |
| Study design | 4 | Present key elements of study design early in the paper | 2 |
| Setting | 5 | Describe the setting, locations, and relevant dates, including periods of recruitment, exposure, follow-up, and data collection | 2 |
| Participants | 6 | (*a*) Give the eligibility criteria, and the sources and methods of selection of participants. Describe methods of follow-up<br>(*b*) For matched studies, give matching criteria and number of exposed and unexposed | 2 |
| Variables | 7 | Clearly define all outcomes, exposures, predictors, potential confounders, and effect modifiers. Give diagnostic criteria, if applicable | 2&3 |

| | | | |
|---|---|---|---|
| Data sources/ measurement | 8* | For each variable of interest, give sources of data and details of methods of assessment (measurement). Describe comparability of assessment methods if there is more than one group | 2&3 |
| Bias | 9 | Describe any efforts to address potential sources of bias | 11&12 |
| Study size | 10 | Explain how the study size was arrived at | 2&3 |
| Quantitative variables | 11 | Explain how quantitative variables were handled in the analyses. If applicable, describe which groupings were chosen and why | 2&3 |
| Statistical methods | 12 | (*a*) Describe all statistical methods, including those used to control for confounding<br>(*b*) Describe any methods used to examine subgroups and interactions<br>(*c*) Explain how missing data were addressed<br>(*d*) If applicable, explain how loss to follow-up was addressed<br>(*e*) Describe any sensitivity analyses | 3 |
| **Results** | | | |
| Participants | 13* | (a) Report numbers of individuals at each stage of study—eg numbers potentially eligible, examined for eligibility, confirmed eligible, included in the study, completing follow-up, and analysed<br>(b) Give reasons for non-participation at each stage<br>(c) Consider use of a flow diagram | 4 |

| | | | |
|---|---|---|---|
| Descriptive data | 14* | (a) Give characteristics of study participants (eg demographic, clinical, social) and information on exposures and potential confounders<br>(b) Indicate number of participants with missing data for each variable of interest<br>(c) Summarise follow-up time (eg, average and total amount) | 4-6 |
| Outcome data | 15* | Report numbers of outcome events or summary measures over time | 8-10 |
| Main results | 16 | (*a*) Give unadjusted estimates and, if applicable, confounder-adjusted estimates and their precision (eg, 95% confidence interval). Make clear which confounders were adjusted for and why they were included<br>(*b*) Report category boundaries when continuous variables were categorized<br>(*c*) If relevant, consider translating estimates of relative risk into absolute risk for a meaningful time period | 9-10 |
| Other analyses | 17 | Report other analyses done—eg analyses of subgroups and interactions, and sensitivity analyses | 10 |
| **Discussion** | | | |
| Key results | 18 | Summarise key results with reference to study objectives | 11 |
| Limitations | 19 | Discuss limitations of the study, taking into account sources of potential bias or imprecision. Discuss both direction and magnitude of any potential bias | 12 |
| Interpretation | 20 | Give a cautious overall | 12 |

| | | | |
|---|---|---|---|
| | | interpretation of results considering objectives, limitations, multiplicity of analyses, results from similar studies, and other relevant evidence | |
| Generalisability | 21 | Discuss the generalisability (external validity) of the study results | 12 |
| **Other information** | | | |
| Funding | 22 | Give the source of funding and the role of the funders for the present study and, if applicable, for the original study on which the present article is based | 12 |

*Give information separately for exposed and unexposed groups.

## Reference

1. Tonelli, M.; Wiebe, N.; Fortin, M.; Guthrie, B.; Hemmelgarn, B.R.; James, M.T.; Klarenbach, S.W.; Lewanczuk, R.; Manns, B.J.; Ronksley, P. Methods for identifying 30 chronic conditions: application to administrative data. *BMC medical informatics and decision making* **2015**, *15*, 31.
